# Loss of Slc26a9 anion transporter alters intestinal electrolyte and HCO_3_^-^ transport and reduces survival in CFTR-deficient mice

**DOI:** 10.1007/s00424-014-1543-x

**Published:** 2014-06-27

**Authors:** Xuemei Liu, Taolang Li, Brigitte Riederer, Henrike Lenzen, Lisa Ludolph, Sunil Yeruva, Biguang Tuo, Manoocher Soleimani, Ursula Seidler

**Affiliations:** 1 Department of Gastroenterology, Hannover Medical School, Hannover, Germany; 2 Department of Gastrointestinal Surgery, Affiliated Hospital of Zunyi Medical College, Zunyi, People’s Republic of China; 3 Department of Gastroenterology, Affiliated Hospital of Zunyi Medical College, Zunyi, People’s Republic of China; 4 Center on Genetics of Transport and Epithelial Biology, University of Cincinnati, Cincinnati, OH USA

**Keywords:** Bicarbonate secretion, Intestine, Anion channel, Anion exchanger, Stomach

## Abstract

Slc26a9 is an anion transporter that is strongly expressed in the stomach and lung. Slc26a9 variants were recently found associated with a higher incidence of meconium ileus in cystic fibrosis (CF) infants, raising the question whether Slc26a9 is expressed in the intestine and what its functional role is. Slc26a9 messenger RNA (mRNA) was found highly expressed in the mucosae of the murine and human upper gastrointestinal tract, with an abrupt decrease in expression levels beyond the duodenum. Absence of SLC26a9 expression strongly increased the intestinally related mortality in cystic fibrosis transmembrane conductance regulator (CFTR)-deficient mice. Proximal duodenal J_HCO3_^−^ and fluid secretion were reduced in the absence of Slc26a9 expression. In the proximal duodenum of young Slc26a9 KO mice, the glands and villi/crypts were elongated and proliferation was enhanced. This difference was lost with ageing, as were the alterations in fluid movement, whereas the reduction in J_HCO3_^-^ remained. Laser dissection followed by qPCR suggested Slc26a9 expression to be crypt-predominant in the duodenum. In summary, deletion of Slc26a9 caused bicarbonate secretory and fluid absorptive changes in the proximal duodenal mucosa and increased the postweaning death rates in CFTR-deficient mice. Functional alterations in the duodenum were most prominent at young ages. We assume that the association of meconium ileus and Slc26a9 variants may be related to maldigestion and impaired downstream signaling caused by loss of upper GI tract digestive functions, aggravating the situation of lack of secretion and sticky mucus at the site of obstruction in CF intestine.

**Electronic supplementary material:**

The online version of this article (doi:10.1007/s00424-014-1543-x) contains supplementary material, which is available to authorized users.

## Introduction

Slc26a9 (solute carrier family 26 member 9) is a member of the Slc26 family of anion transporters with strong expression at apical and intracellular membranes of the lung and stomach [25, 48]. In addition, Slc26a9 is expressed in specialized cells in the kidney, the neural system, and the reproductive tract [1, 8, 14]. Oocyte and human embryonic kidney (HEK) cell expression studies resulted in conflicting data regarding the Slc26a9 transport function. Dorwart et al. suggested SLC26a9 to be a highly selective Cl^-^ channel with minimal OH^-^/HCO_3_^-^ permeability that is regulated by the WNK kinases [14]. Loriol et al. described an enhanced conductivity of Slc26a9 by high HCO_3_^-^ concentrations [26], while other studies suggest it to operate both as a Cl^-^ channel or a Cl^-^/HCO_3_^-^ exchanger [13, 47] with a possible Na^+^(cation) dependence [8]. In addition, an interaction with the cystic fibrosis transmembrane conductance regulator (CFTR) is suggested by several in vitro studies [4, 6, 9, 26, 29], but conflicting data exist as to whether this interaction is inhibitory or stimulatory.

We previously reported an expression of Slc26a9 in gastric parietal cells, and a progressive postnatal loss of parietal cells and acid secretory capacity, resulting in achlorhydria by 6 weeks of age in Slc26a9-deficient mice [48]. While this, at first glance, suggested Slc26a9 to be the long-sought apical anion conductance through which the Cl^-^ ions pass and are co-secreted with the protons during gastric acid secretion, the fact that newborn mice had normal acid secretory rates argues for caution with this conclusion. In the airways, Slc26a9 deletion resulted in loss of the short-circuit current (I_sc_) increase which was seen in the bronchi excised from mice that have been subjected to IL-13-mediated airway inflammation [2], whereas the I_sc_ response to cAMP- and Ca^2+^-dependent stimulation was not affected [3]. While unequivocally demonstrating the physiological and pathophysiological relevance of Slc26a9 expression in the two organs with the highest Slc26a9 expression, the role and regulation of Slc26a9 in epithelial transport remained mysterious.

Recently, polymorphisms in the *slc26a9* gene have been found to be associated with an increased risk for meconium ileus in infants with cystic fibrosis [41]. We therefore undertook the present study, which searches for Slc26a9 expression in the entire murine and human gastrointestinal tract and determines the cellular location of Slc26a9 expression in the small intestine. Because Slc26a9 expression was much higher in the proximal than the distal duodenum, yet the tissue morphology is very similar, we studied epithelial transport function in the proximal and distal duodenum in Slc26a9 wild-type (WT) and knock-out (KO) mice to get more insight into the role of this transporter in the transport function of a highly complex intestinal epithelium.

## Materials and methods

### Animals

The *Slc26a9*-deleted mouse strain, whose establishment and characteristics have been described elsewhere [48], was congenic on the S129/svj background and was bred and genotyped in accordance with the Institutional Animal Care and Use Committee (IACUC) at Hannover Medical School. For isolated experiments, *Slc26a3*-deleted mice, congenic on the C57/B6 background, were used, which had been bred as recently described [46]. The Slc26a3 KO and WT littermates remained cohoused after genotyping (the heterozygotes were removed), and both received the identical special diet and drinking fluid [46]. We also bred CFTR KO and WT mice as previously described [45] and CFTR/Slc26a9 double-deficient mice, which were a mixed FVB/N and S129/svj background. The mice were age- and sex-matched and used between 3 weeks and 6 months of age (studied at different ages). All experiments involving animals were approved by the Hannover Medical School committee on investigations involving animals and an independent committee assembled by the local authorities.

### Patient biopsy acquisition

Informed consent was obtained prior to endoscopy. In addition to the biopsies for routine histology, an additional biopsy was taken from the fundus, antrum, and duodenum of patients that underwent upper GI endoscopy or from different parts of the colon (sigmoid, transverse, and proximal) as well as the terminal ileum of patients that underwent lower GI endoscopy. We selected patients with a healthy upper or lower digestive tract, respectively (neither macroscopic nor microscopic changes). The biopsy was rinsed in phosphate-buffered saline, immediately transferred to RNA*later®* (Life Technologies) and homogenized. The protocols were approved by the Hannover Medical School Ethics Committee.

### Quantitative real-time PCR

Mucosa was scraped from the different intestinal segments from WT mice and immediately homogenized in RNA*later*, as described previously [44]. RNA was isolated from scraped proximal and distal duodenal mucosa from WT mice using Nucleo Spin RNAII Total RNA Isolation Kit (Macherey and Nagel, Düren, Germany). Reverse transcription was performed with SuperScriptIII RNase H-Reverse Transcriptase (Invitrogen, Karlsruhe, Germany). The primer sequence of different genes is shown in Supplementary Table 1. We used actin as the reference gene for the Slc26a9 expression in the different intestinal segments, because it is expressed in an epithelial-predominant fashion in all intestinal segments (in the absence of inflammatory cell infiltration).

### Laser capture microdissection and mRNA quantification

For microdissection of crypt and villous duodenocytes from Slc26a9 WT mice, tissue collection and fixation were performed as described by Goldworthy et al. [18]. The laser microdissection microscope (MMI CellCut Plus Microdissection System, Molecular Machines & Industries, Glattburg, Switzerland) was used to microdissect duodenocytes from the cryptal and the upper villous region (not the tip). Approximately 3,000 microdissected cells per region (crypt versus villi from each duodenum) were picked into RLT buffer (Qiagen, Hilden, Germany), RNA was isolated with RNeasy Microkit (Qiagen), and the reverse transcription and the PCR amplification were performed as described previously [34].

### Survival statistics for Slc26a9 KO and WT, CFTR KO and WT, and the double-deficient mice

Survival statistics were performed for mice after the weaning period, by following spontaneous death over a period of up to 1 year. Within a short time span after death, macropathological examination was performed to check for the occurrence of intestinal obstruction, which was the cause of death in CFTR-deficient mice after weaning. The incidence rate of this complication can be lowered to but not fully prevented by dietary measures. Occasional long-term survivors also displayed tumor formations as presumed causes of death, but we did not study this age period in the current study.

### Ussing chamber experiments

Ussing chamber experiments were performed exactly as described previously [45]. The serosal solution contained (in mM) 108 NaCl, 25 NaHCO_3_, 3 KCl, 1.3 MgSO_4_, 2 CaCl_2_, 2.25 KH_2_PO_4_, 8.9 glucose, and 10 sodium pyruvate and was gassed with 95 % O_2_/5 % CO_2_ (pH 7.4). The mucosal solution (154 mM NaCl) was gassed with 100 % O_2_. Basal parameters were measured for 30 min, forskolin (FSK) plus IBMX were then added to the serosal solution with an end concentration of 10 μM plus 100 μM, and J_HCO3_^-^ was continuously titrated for the subsequent 30 min. J_HCO3_^-^ was calculated for 5-min intervals.

### Mouse anesthesia

Mouse anesthesia and control of body functions have recently been described in detail [46]. Briefly, body temperature was maintained at 37.5 °C by means of a heating pad and a rectal thermistor probe; blood pressure was monitored via a catheter placed in the left carotid artery, which was also used for continuous infusion of an isotonic Na_2_CO_3_ solution and isotonic Ringer’s solution to maintain acid/base and fluid balance, which was assessed by periodic blood gas measurements.

### Experimental protocol for the measurement of proximal and distal duodenal J_HCO3_^-^ and fluid absorption in vivo

The surgical procedure for the measurement of duodenal J_HCO3_^-^ in anesthetized mice has been described in detail [33], but for this project, it was performed with the following modifications: for proximal duodenal perfusion, a small polyethylene tube (PE100, inner diameter 1 mm) with a distal flange was inserted through a hole made in the forestomach, guided through the pylorus, and secured by a ligature directly in the pylorus. The outlet tube (PE200, inner diameter 2 mm) was put through the middle duodenum and fixed at 1 cm distal to the pylorus. For the distal duodenum, the inlet tube was induced into the middle duodenum and secured about 2 cm distal to the pylorus. The effluent tube was induced into the jejunum and secured near the Treitz ligament. The pancreatic/biliary duct was ligated near the entry into the intestine. Each perfused segment (proximal and distal) was approximately 1 cm in length, and the exact length was measured after excising the segment after sacrifice and measuring the length without stretching. J_HCO3_^-^ was determined for each 20-min period, and J_HCO3_^-^ is expressed as micromoles of base secreted per centimeter of intestine per hour (μM cm^−1^ h^−1^). The measurement of fluid absorption was performed as described previously [46]. The composition of perfusate solutions were as follows: 154 mM NaCl + 0.2 % dimethyl sulphoxide (DMSO) and 154 mM NaCl with 20 mM FSK in 0.2 % DMSO. This concentration of DMSO was found to be the maximum concentration that did not alter duodenal fluid absorption.

### Histological and immunohistochemical study of the intestinal tract

Mice were sacrificed, and the different intestinal segments were excised, fixed with 4 % paraformaldehyde, embedded in paraffin; tissue sections (2 μm) were prepared, deparaffinized, and stained with hematoxylin and eosin by standard protocols. Antigen Ki67 staining was performed according to the suggestions of the manufacturer (Vector Labs, Biozol, Eching, Germany).

### Agents

Forskolin and 3-isobuty1-1-methlxanthine (IBMX) were purchased from Alexis Biochemicals (Lörrach, Germany). Tetrodotoxin was purchased from Biotrend Chemicals AG (Wengen, Switzerland). Other reagents were purchased from Sigma-Aldrich (Deisenhofen, Germany) or where specifically mentioned in the “Materials and methods” section.

### Statistical evaluation

All results were expressed as mean ± SEM, “J_HCO3_^-^” and “I_sc_” represent the mean value of basal HCO_3_^-^ secretion and I_sc_, respectively, “ΔJ_HCO3_^-^” represents, for each experiment, the maximal response after secretagogue addition averaged over the basal value. The data were analyzed by one-way ANOVA if for multiple comparisons or student’s *t* test for paired samples. Results were considered significant at *P* < 0.05.

## Results

### mRNA expression of Slc26a9 and CFTR in the different segments of murine and human gastrointestinal tract

**Fig. 1:**
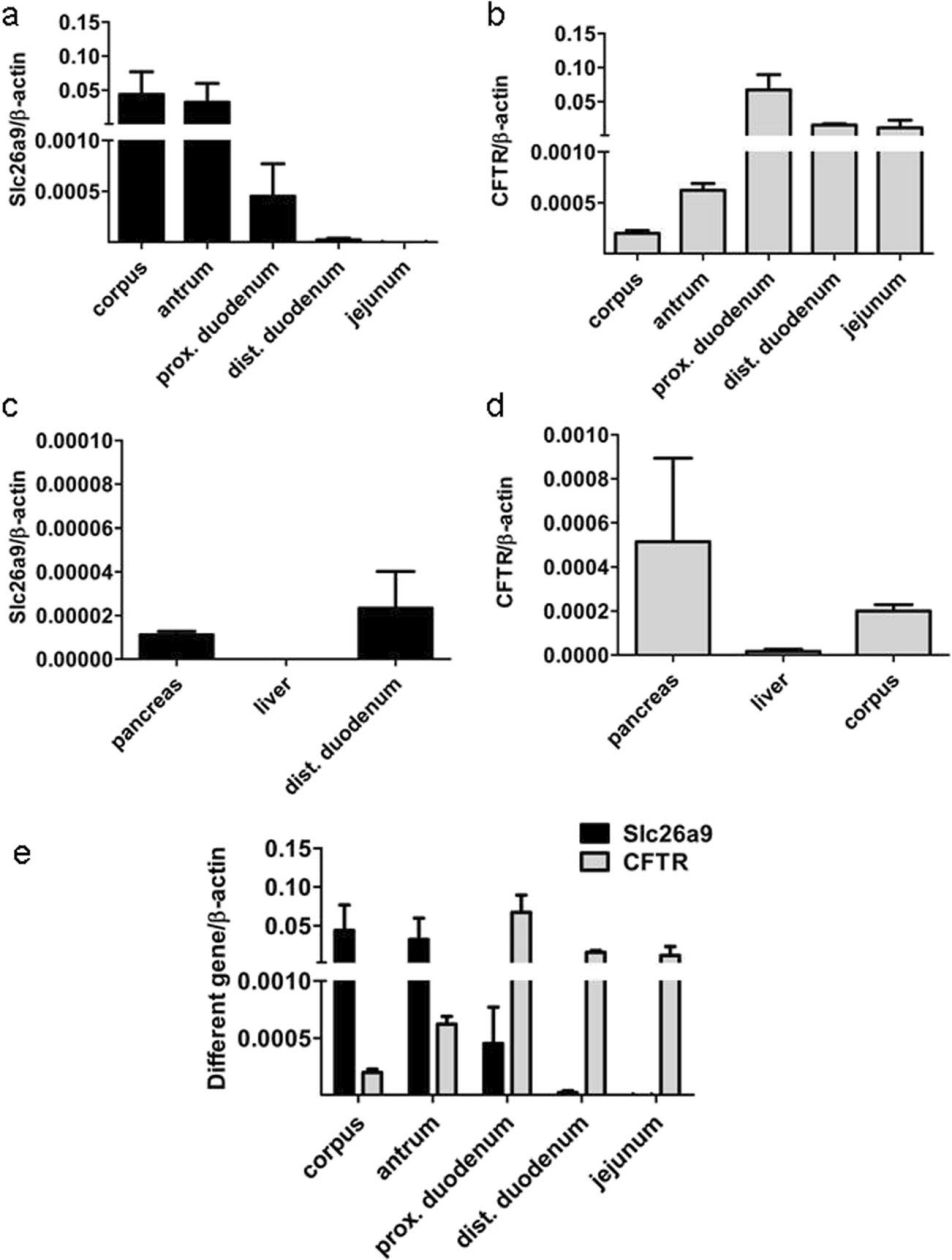
mRNA expression of Slc26a9 and CFTR in the murine GI tract. Slc26a9 is predominately expressed in the stomach, to some extent in proximal duodenum, low in the pancreas, and not expressed in the liver and the more distal GI tract (**a**, **c**). In contrast, CFTR expression is low in the stomach, pancreas, and liver but higher in the intestine (**b**, **d**). **e** Comparison of mRNA expression between Slc26a9 and CFTR in the different segments of GI tract, using the same control gene and tissue sample. *n* = 6–9

We compared the expression of Slc26a9 with that of the other important intestinal Cl^-^ channel, namely CFTR. Slc26a9 was strongly expressed in the stomach, and to some extent the proximal duodenum but not in the more distal GI tract (Fig. 1a). In contrast, CFTR expression was low in the stomach, pancreas, and liver but high in the intestine (Fig. 1b, d). Figure 1e has shown both genes in the same graph, using the same *y* axis. Low Slc26a9 expression was also found in the pancreas, while the liver did not reveal specific Slc26a9 messenger RNA (mRNA) expression (Fig. 1c).

**Fig. 2:**
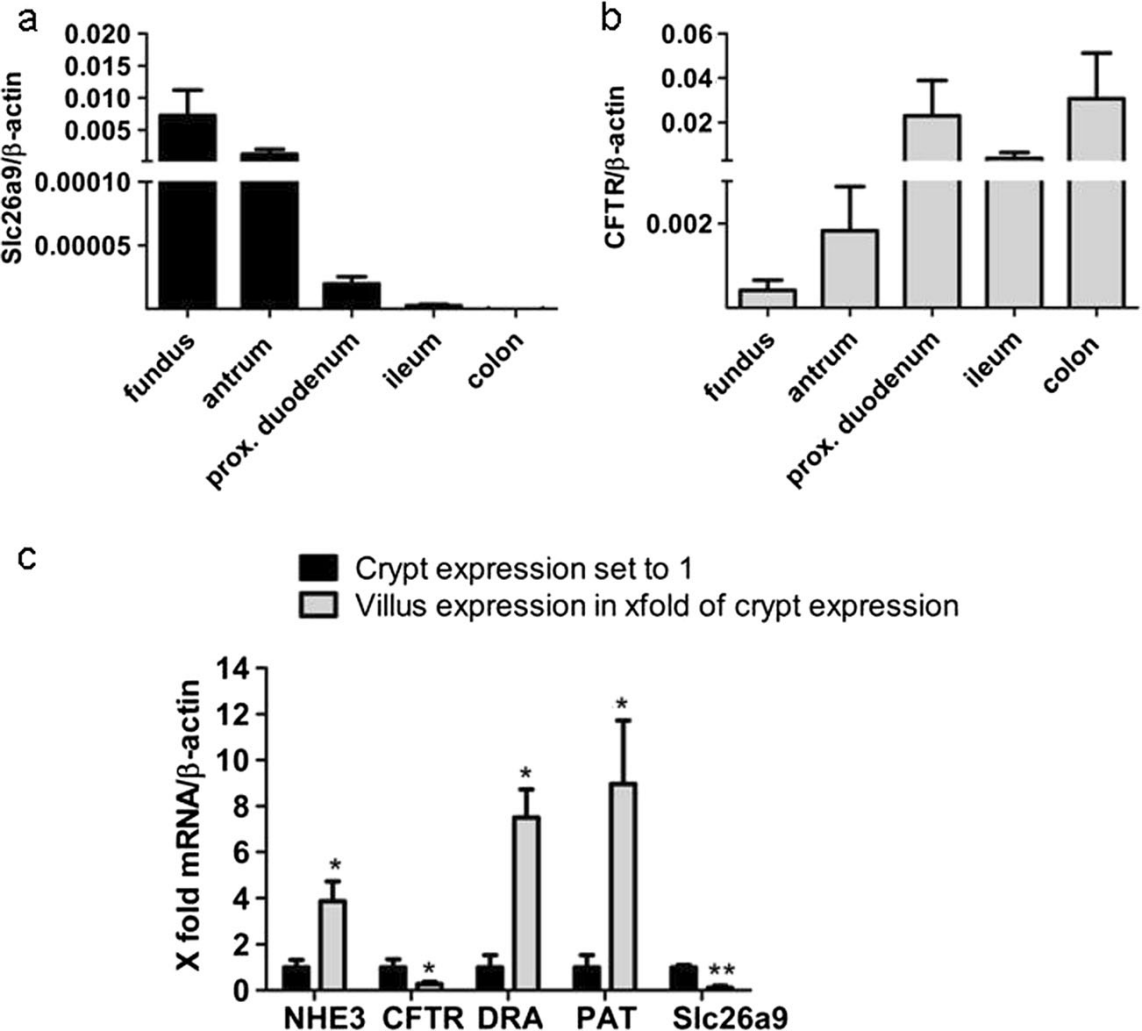
mRNA expression of Slc26a9 and CFTR in human biopsies, and crypt-villus gradient of transporter expression in laser-dissected murine proximal duodenal enterocytes. The expression pattern of Slc26a9 and CFTR mRNA in human and murine GI tract is similar. **a** Slc26a9 is strongly expressed in the fundus/corpus and antrum region but with lower levels in proximal duodenum, very low in ileum, and not expressed in the colon. *n* = 6-8. In contrast, **b** CFTR expression is low in the fundus/corpus and antrum but higher in the intestine including proximal duodenum, ileum, and colon. *n* = 6–8. **c** The cell-specific expression location of different ion transporters was measured by qPCR in laser-dissected villi and crypt of murine proximal duodenal epithelium. The cryptal expression was set to 1; the villus expression is given as a multiple of cryptal expression. Slc26a9 and CFTR appear to be predominantly cryptal-expressed. Slc26a3, Slc26a6, and NHE3 are villus-predominant. *n* = 5-10. *P < 0.05, **P < 0.01

We also studied Slc26a9 and CFTR expression in biopsies taken from human gastric fundus/corpus, antrum, duodenum, ileum, and colon. We found a very high expression of Slc26a9 in both the fundic/corpus and antral biopsies, a much lower expression in the duodenum, an almost negligible expression in the ileum, and no expression in the colon (Fig. 2a). In contrast, CFTR is highly expressed in the proximal duodenum, ileum, and colon but with lower levels in the fundic/corpus and antral biopsies (Fig. 2b). Thus, the segmental expression of Slc26a9 and CFTR in the human gastrointestinal tract is similar to that in the mouse.

### Slc26a9 expression along the duodenal crypt villus axis

Although Slc26a9 displays low expression levels in the duodenum at the whole organ level (Fig. 1a), its function may nevertheless be significant, depending on its localization. We therefore performed laser capture from duodenal cryosections. We separately dissected duodenal crypts and the epithelial cells along the upper-middle part of the villi, without taking the villus tip cells, which are known to undergo apoptosis and do not show strong mRNA expression of transporters when studied by in situ hybridization [20]. For validation, we studied the expression of other transporters with a known crypt-villus expression gradient validated by immunohistochemistry. In Fig. 2c, the mRNA expression of each transporter gene, measured in samples excised from the cryptal areas, was set to one and the expression in the villus preparation as a multiple of the cryptal expression level. Slc26a3 (DRA), Slc26a6 (putative anion transporter 1 (PAT-1)), and sodium-hydrogen exchanger 3 (NHE3) had higher than one villus/crypt expression level, whereas CFTR and Slc26a9 had lower than one villus/crypt expression level, indicating crypt predominance.

### Survival statistics for Slc26a9 KO and WT, CFTR KO and WT, and the double-deficient mice

Slc26a9 KO mice display high mortality 1–2 days after birth (presumably due to pulmonary complications), whereas CFTR KO mice display the highest mortality rate at the time of weaning [30]. To test how the absence of Slc26a9 expression affects the survival of CFTR KO mice, we followed a large series of Slc26a9 KO and WT littermates, CFTR KO and WT littermates, and a small number of double KO mice from postnatal week 3 (in the weaning phase, at the time of genotyping) until death, for a period of 1 year. The CFTR KO as well as the double KO mice had received intense nutritional therapy (started at postnatal week 2).

**Fig. 3:**
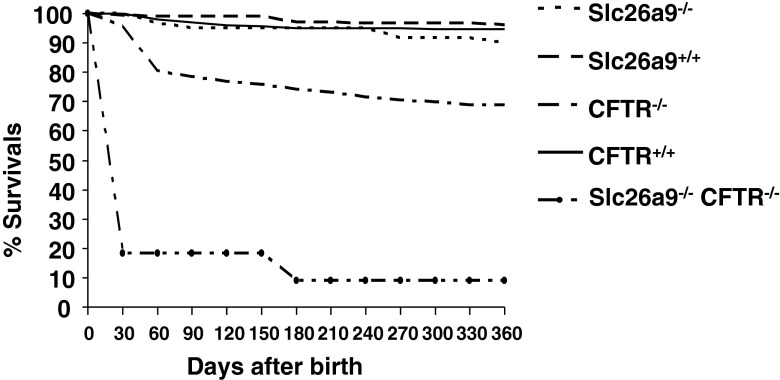
Survival statistics for Slc26a9^+/+^ and Slc26a9^−/−^, CFTR^+/+^ and CFTR^−/−^, and Slc26a9/CFTR double-deficient mice. While the Slc26a9^+/+^, the Slc26a9^−/−^, as well as CFTR^+/+^ mice showed no reduction in survival from approximately 15 days after birth (at the time of genotyping) up to 1 year, the CFTR^−/−^ had a significant decrease in survival rates with the sharpest decline in the postweaning phase. The increased mortality in the weaning phase was strongly enhanced by additional Slc26a9 deletion. Slc26a9^+/+^ mice *n* = 193, Slc26a9^−/−^ mice *n* = 60, CFTR^+/+^ mice *n* = 207, CFTR^−/−^ mice *n* = 112, and Slc26a9/CFTR double-deficient mice *n* = 11

Figure 3 shows the survival statistics from the time of genotyping at approximately 3 weeks of age to approximately 1 year of age. It is clear that once the Slc26a9 KO mice survive to week 3, no significant survival disadvantage is seen compared to WT until week 40, when a very small decrease in survival compared to WT is noted (Fig. 3). This is different in CFTR KO mice, which show a relatively high mortality in the early postweaning phase and a continuing decline in survival compared to WT littermates despite fiber-free food, special bedding, and drinking solution containing polyethylene glycol (PEG). While very old mice (>1 year/age) have been found to develop tumors, in this cohort, the cause of death was intestinal obstruction in those mice where we had performed postmortem analysis, as we and others have described previously [30, 31].

The additional loss of Slc26a9 strongly increased the mortality in the weaning period. Because the majority of mice disappeared before postnatal day 30, during which the litter was still with the parents, we could not ascertain the cause of death in most instances. However, since the double KO mice were indistinguishable from their littermates at the time of genotyping, we believe with a very high level of confidence that the cause of death was, as in the older mice, intestinal obstruction.

### The HCO_3_^-^ secretory rate is higher in proximal than the distal duodenum in vivo and in vitro

**Fig. 4:**
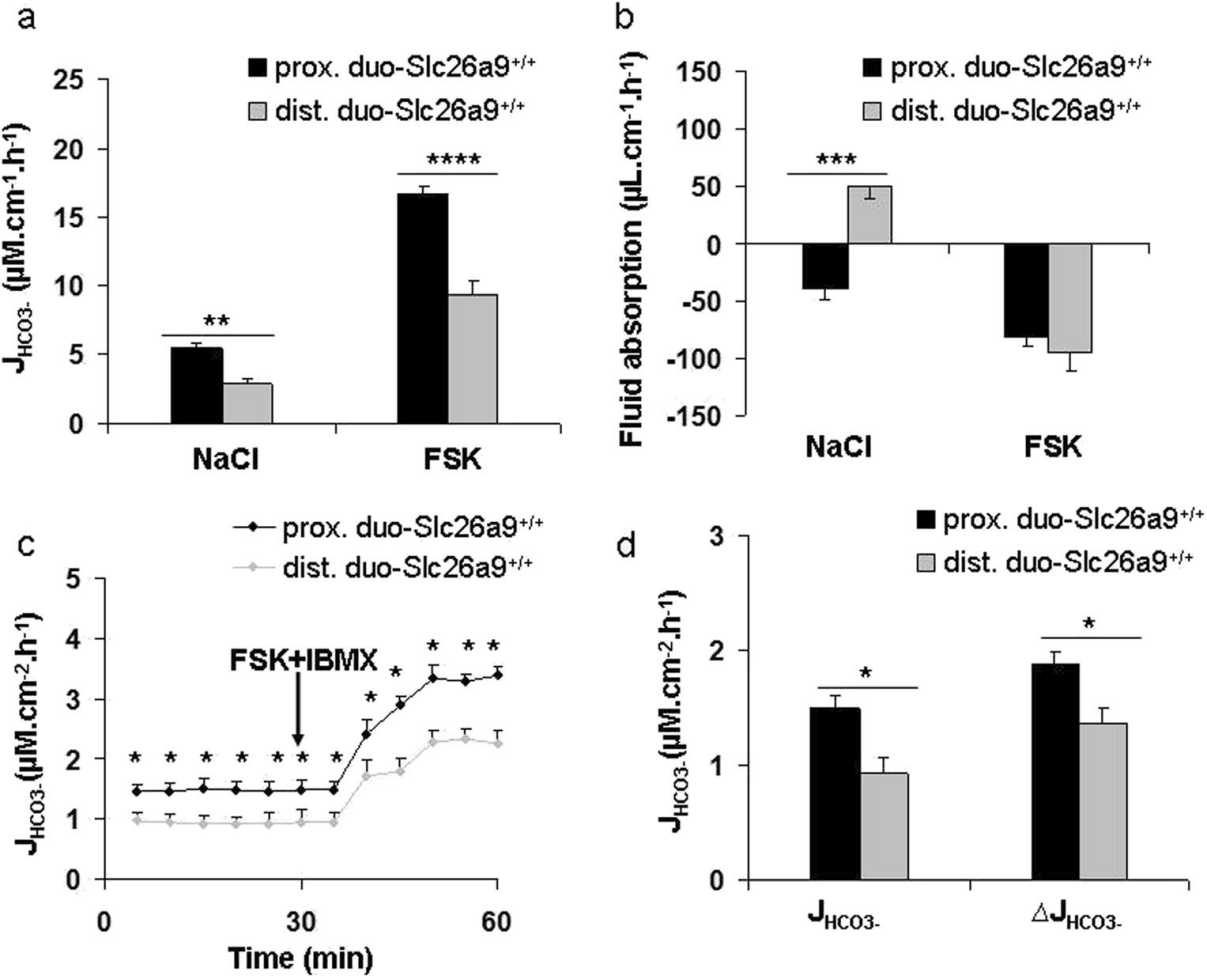
HCO_3_^-^ secretion of proximal and distal duodenum of Slc26a9^+/+^ mice both in vitro and in vivo as well as fluid absorption in vivo. **a** Basal J_HCO3_^-^, as well as FSK-stimulated HCO_3_^-^ secretory rate (∆J_HCO3_^-^) was significantly higher in the proximal duodenum than distal duodenum in anesthetized Slc26a9^**+/+**^ mice. *n* = 6–16. **b** The proximal duodenum secretes fluid in the basal state, whereas the distal duodenum absorbs in the basal state, but the fluid secretory response to FSK was identical. *n* = 6-16. **c** J_HCO3_^-^ as well as FSK-induced ∆J_HCO3_^-^ were also significantly higher in chambered muscle-stripped-isolated proximal versus distal duodenal mucosa. *n* = 5–6. **d** Shows the comparison between basal J_HCO3_^-^ and ΔJ_HCO3_^-^ in proximal and distal duodenum. *n* = 5-6, *P < 0.05, **P < 0.01, ***P < 0.001, ****P < 0.0001

HCO_3_^-^ secretory rates and the transepithelial fluid movements were assessed in the proximal and distal duodenum of anesthetized WT mice. A significantly higher HCO_3_^-^ secretory rate both in the basal state (NaCl as luminal fluid) and during FSK stimulation (FSK in luminal fluid) was observed in the proximal (first 1 cm of duodenum) than the distal duodenum (last 1 cm before ligament of Treitz) (Fig. 4a) in vivo. Fluid movement was also assessed in the proximal and the distal duodenum. Interestingly, the proximal duodenum secretes fluid in the basal state, whereas the distal duodenum absorbs in the basal state. The HCO_3_^-^ secretory response to FSK was stronger in the proximal than the distal duodenum, whereas the fluid secretory response to FSK was identical (Fig. 4b).

The basal and FSK-stimulated HCO_3_^-^ secretory rates were also measured in muscle-stripped mucosa of the same segments of WT duodenum in Ussing chamber setups. When studied simultaneously from the same set of mice, proximal duodenal mucosa had a higher basal and FSK-stimulated J_HCO3_^-^ than the distal mucosa (Fig. 4c–d), whereas no significant differences were seen in I_sc_, FSK-induced ∆I_sc_, PD, and *R*_t_ (Table 1).

**Table 1:** Electrical parameters of different segments of intestine in Slc26a9^+/+^ and Slc26a9^−/−^ mice

	PD (mv)		*R*_t_ (Ώ cm^2^)		I_sc_ (μeq cm^−2^ h^−1^)		
	Basal	Peak FSK + IBMX	Basal	Peak FSK + IBMX	Basal	Peak FSK + IBMX	∆I_sc_
Proximal duodenum Slc26a9^+/+^	0.94 ± 0.36	4.22 ± 0.65	29.05 ± 3.92	34.00 ± 4.15	1.19 ± 0.33	6.27 ± 0.58	5.07 ± 0.42
Proximal duodenum Slc26a9^−/−^	0.80 ± 0.28	4.06 ± 0.52	35.60 ± 9.91	41.4 ± 11.57	1.70 ± 0.45	6.93 ± 1.03	5.22 ± 0.59
Distal duodenum Slc26a9^+/+^	0.78 ± 0.21	4.52 ± 0.70	30.40 ± 2.37	33.96 ± 2.47	1.29 ± 0.26	5.89 ± 0.72	4.60 ± 0.61
Distal duodenum Slc26a9^−/−^	0.46 ± 0.16	3.38 ± 0.39	27.80 ± 3.91	28.80 ± 3.78	0.83 ± 0.28	5.12 ± 0.91	4.28 ± 0.65

Short-circuit current (I_sc_), potential difference (PD), and electrical resistance (*R*_t_) of proximal duodenum and distal duodenum in basal state and peak stimulation as well as net I_sc_ response after stimulation both in Slc26a9^+/+^ and Slc26a9^−/−^ mice. *n* = 5–6

### Slc26a9 deletion reduced J_HCO3-_ in the proximal but not the distal duodenum

**Fig. 5:**
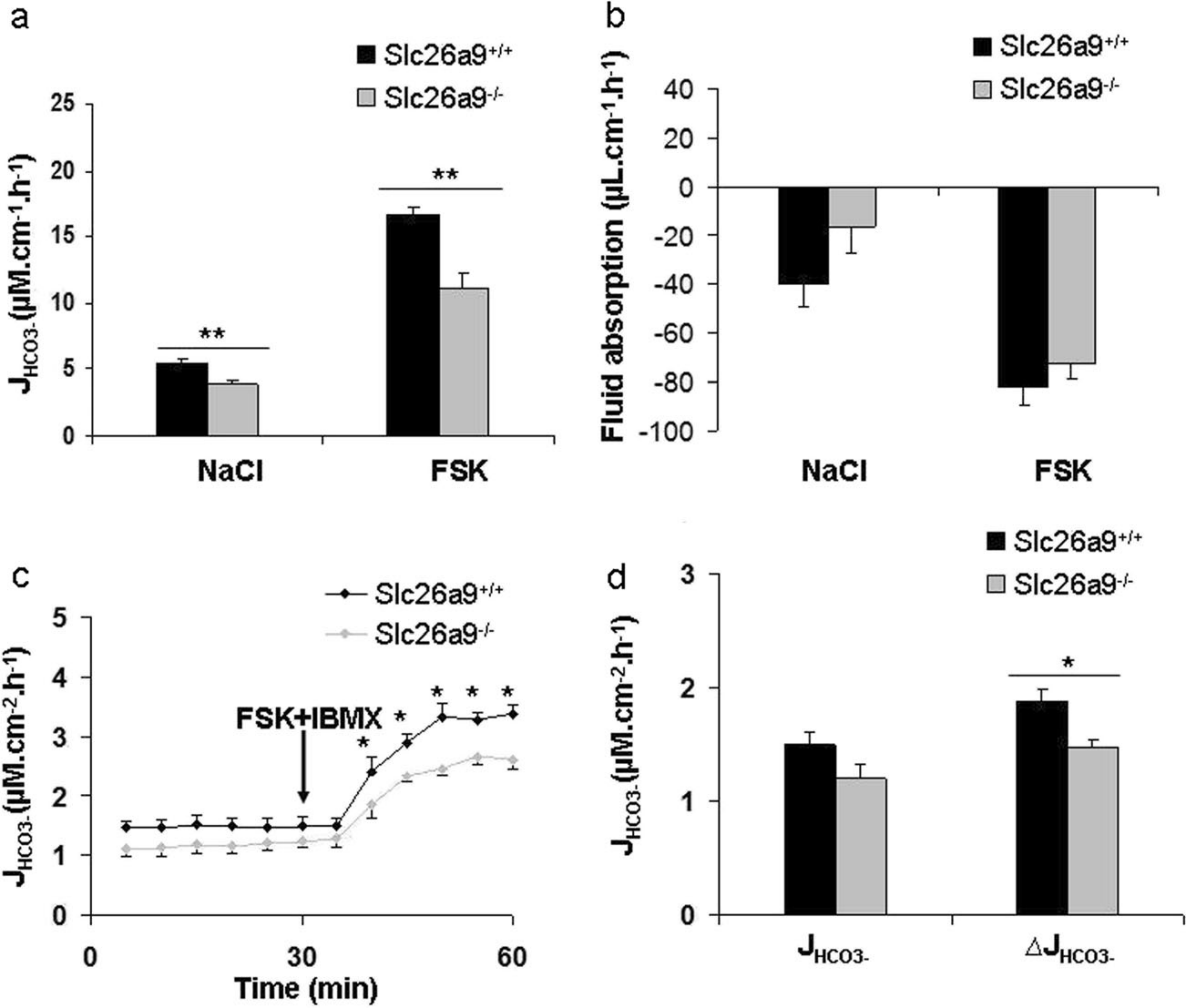
Proximal duodenal HCO_3_^-^ secretion is altered in the absence of Slc26a9. **a** Reduced basal J_HCO3_^-^, as well as FSK -induced ∆J_HCO3_^-^, in the proximal duodenum of anesthetized Slc26a9^−/−^compared to WT mice. **b** Fluid transport rates were not statistically different in the investigated mouse cohort. *n* = 16. **c** Lower FSK - stimulated ∆J_HCO3_^-^ in the isolated proximal duodenal mucosa of Slc26a9^−/−^compared to WT mice in vitro. *n* = 5-6. **d** Represents the basal J_HCO3_^-^, and the FSK  +  IBMX -induced ∆J_HCO3_^-^. *n* = 5–6, *P  < 0.05, **P  < 0.01

We next studied the same parameters in the proximal and distal duodenum of anesthetized Slc26a9 KO and WT mice, respectively. A significant reduction in basal as well as FSK-stimulated J_HCO3_^-^ was observed in the proximal (Fig. 5a) but not the distal duodenum of Slc26a9 KO mice (Supplementary Fig. 1a, b), whereas the difference in fluid secretion of the proximal duodenum did not reach statistical significance (Fig. 5b).

The HCO_3_^-^ secretory parameters were also studied in the isolated proximal as well as the distal duodenal mucosa from Slc26a9 KO and WT mice, respectively. A significantly reduced FSK-stimulated J_HCO3_^-^ was observed in the proximal duodenum from Slc26a9 KO compared to WT mucosa; the basal J_HCO3_^-^ was also lower, but this did not reach statistical significance (Fig. 5c, d). I_sc_, FSK-induced ∆I_sc_, PD, and *R*_t_ were not different (Table 1). In the distal duodenum, there was no difference between Slc26a9 and WT mucosa in any parameter (Supplementary Fig. 1c, d). The experiments demonstrate that the difference in HCO_3_^-^ secretion between Slc26a9 KO and WT is, at least in part, of epithelial origin.

### The HCO_3_^-^ secretory defect in Slc26a9 KO duodenum is associated with young age

**Fig. 6:**
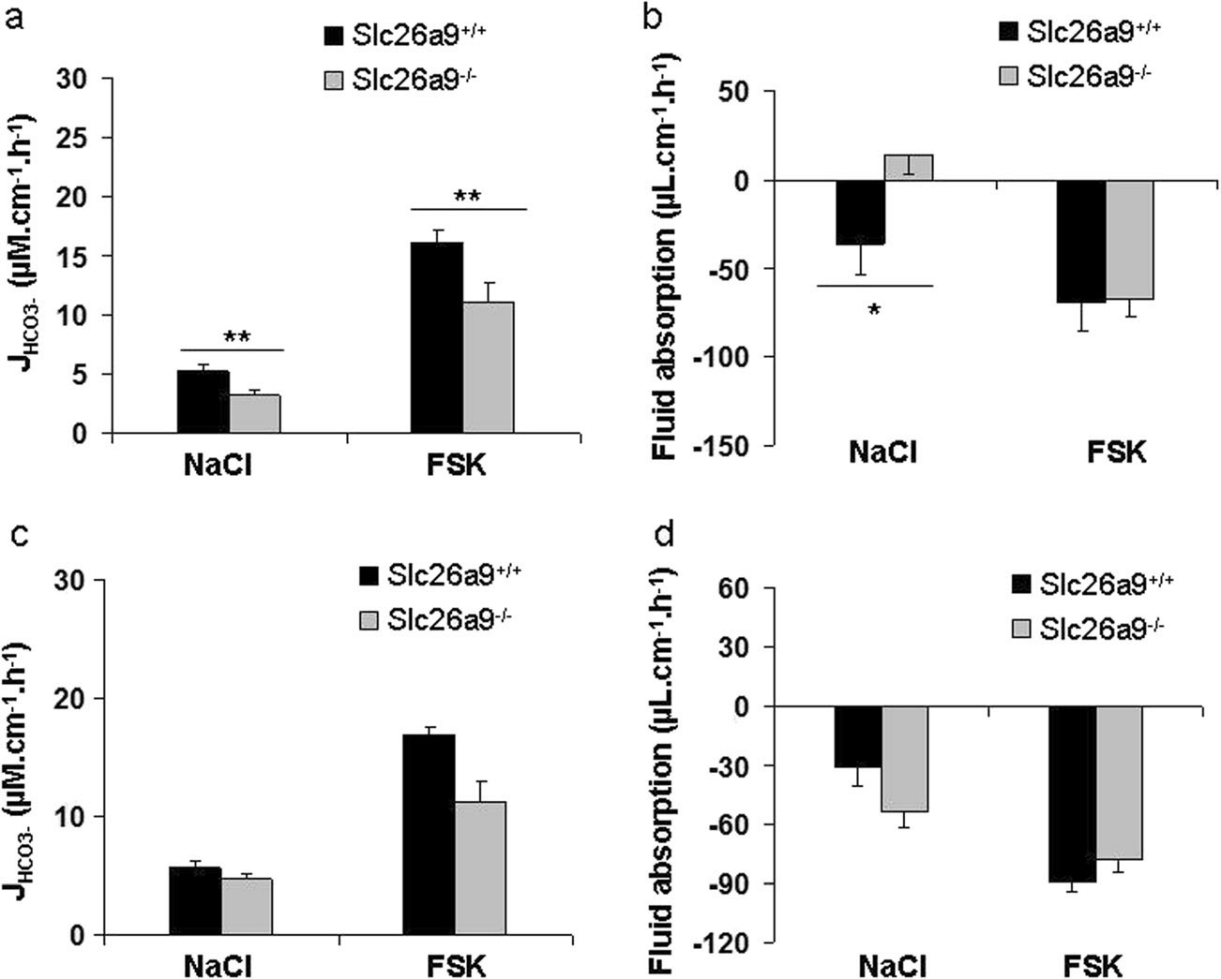
HCO_3_^-^ secretion and fluid absorption in the proximal duodenum of Slc26a9^−/−^ and WT mice at different age. **a** The proximal duodenum of young (3–6 weeks, <20 g bodyweight) Slc26a9^−/−^ mice displayed significantly lower HCO_3_^-^ secretory rates both at basal state and after FSK stimulation and was in a fluid absorptive state, whereas the proximal duodenum of the WT was in a secretory state. **b** FSK-induced fluid secretion was not different between the genotypes. *n* = 6–9. However, such a difference in basal fluid absorption was not observed in mice above 4 months of age (**c-d**). *n* = 7-10. *P < 0.05, **P < 0.01

The original experiments described above were performed in adulthood and over a fairly large age range from 6 weeks to 6 months. When we stratified mice for age and weight, we observed that the difference in proximal duodenal ion transport parameters was large at young ages (3-4 weeks, <20 g bodyweight) (Fig. 6a, b) and was not significant any more in mice above 4 months of age (Fig. 6c, d). In the young mice, the basal as well as FSK-stimulated J_HCO3_^-^ was significantly lower in the Slc26a9 KO than WT proximal duodenum (Fig. 6a). In addition, the proximal duodenum was in a fluid absorptive state in Slc26a9 KO mice, as compared to a secretory state in WT mice (Fig. 6b). In the ageing mice group, these differences were either completely abolished or did not reach the level of significance anymore (Fig. 6c–d).

### The proximal duodenal mucosa of Slc26a9 KO mice displays cryptal and villi elongation and a longer proliferative zone

**Fig. 7:**
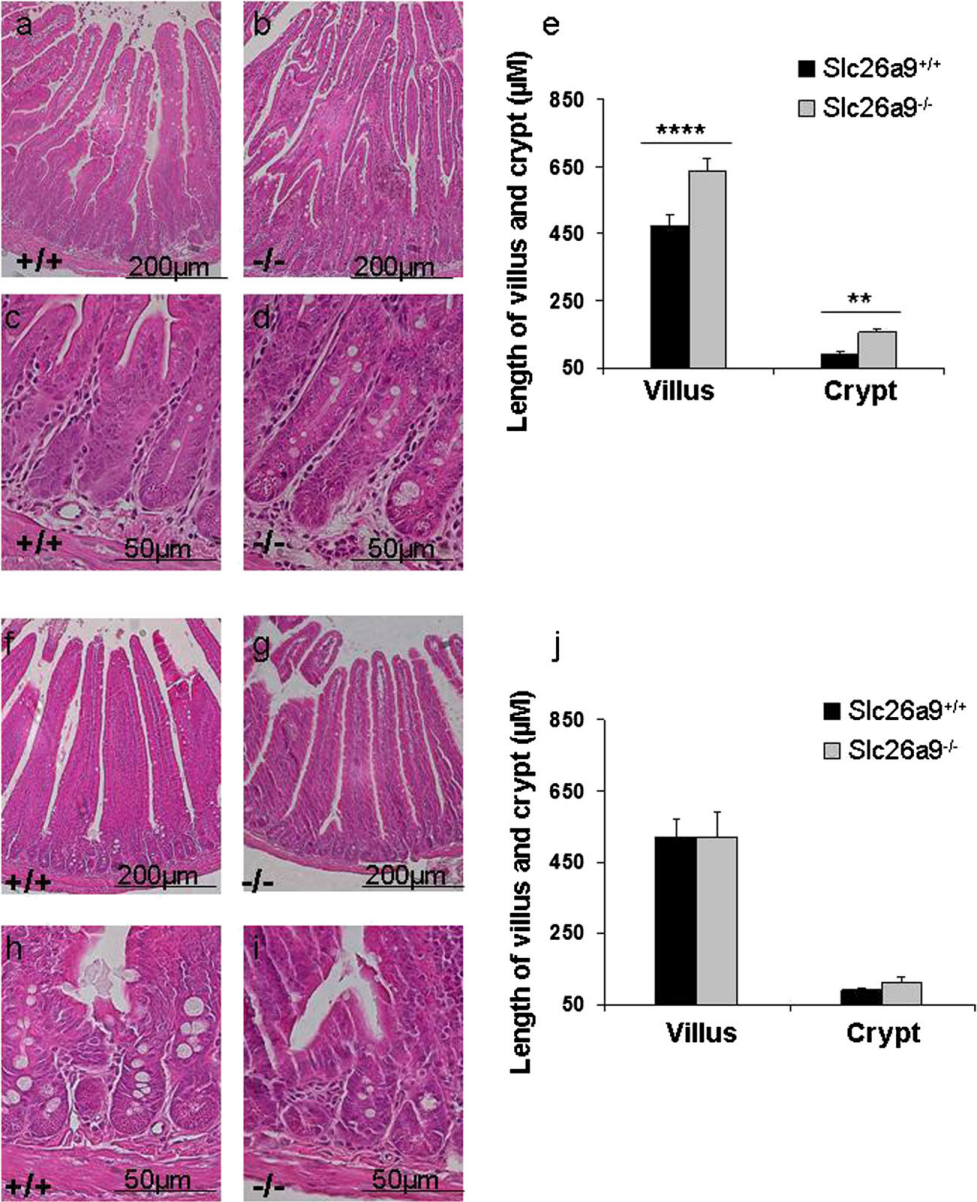
Histological analysis of proximal duodenum in *Slc26a9-*deficient mice at young age. The proximal duodenum of Slc26a9^−/−^ mice displayed elongated crypts and villi (**b**, **d**) when compared with WT mice (**a**, **c). c**, **d** Crypt region with higher magnification. This difference was not seen in old mice (**f-i**). **h**, **i** Crypt region with higher magnification. Measurement of length in crypts and villi in Slc26a9^+/+^ and Slc26a9^−/−^ mice at young and old age (**e**, **j**). *Scale bars* 200 and 50 μm. *n* = 5–6. **P < 0.01, ***P < 0.001

To search for morphological correlates of the observed functional changes, we performed morphometry of the proximal duodenum in both young (3-6 weeks) and older (above 4 months) Slc26a9 KO and WT mice. The measurement of the duodenal villus and crypt lengths of the proximal duodenum at these two ages revealed, surprisingly, that the crypts and villi were significantly longer in the young Slc26a9 KO than WT mice (Fig. 7a-e), but that this difference disappeared in aged mice (Fig. 7f-j). In addition, no difference was observed in other segments of the intestine (Supplementary Fig. 2).

**Fig. 8:**
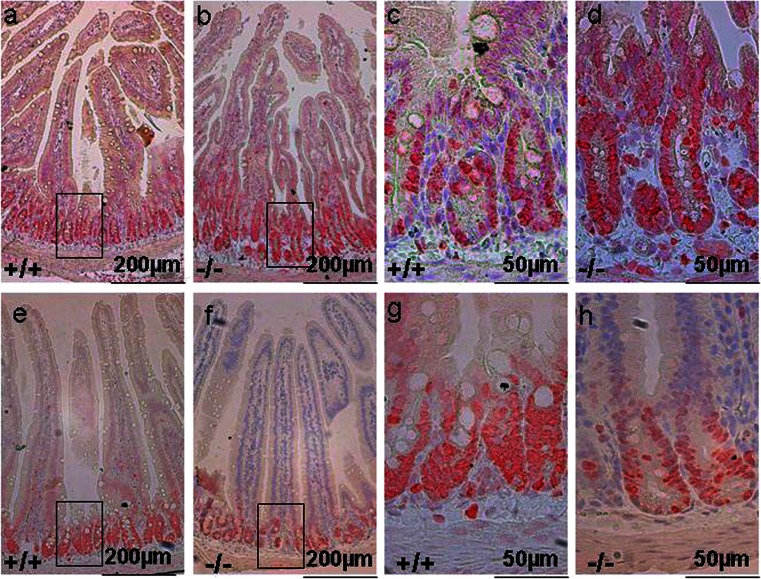
The proliferative zone is longer in the proximal duodenum of Slc26a9^−/−^ (**b**, **d**), with intense labelling of many cells with Ki67 antibody, compared to WT (**a**, **c**) mice. **c**, **d** are the inserts from **a**, **b** with higher magnification. This difference was not observed in old mice (**e**–**h**). **g**, **h** The *inset* of e and f with higher magnification. *Scale bars* 200 and 50 μm. *n* = 5-6

The proliferative zone was stained with an anti-Ki67 antibody. A longer proliferative zone was observed with intense labeling for Ki67 in many cells, in the duodenum of young mice (Fig. 8a-d). This difference disappeared at old age (Fig. 8e-h).

### Absence of Slc26a3 (DRA) causes a reduction in FSK-induced ∆J_HCO3_^-^ in the distal but not the proximal duodenum

**Fig. 9:**
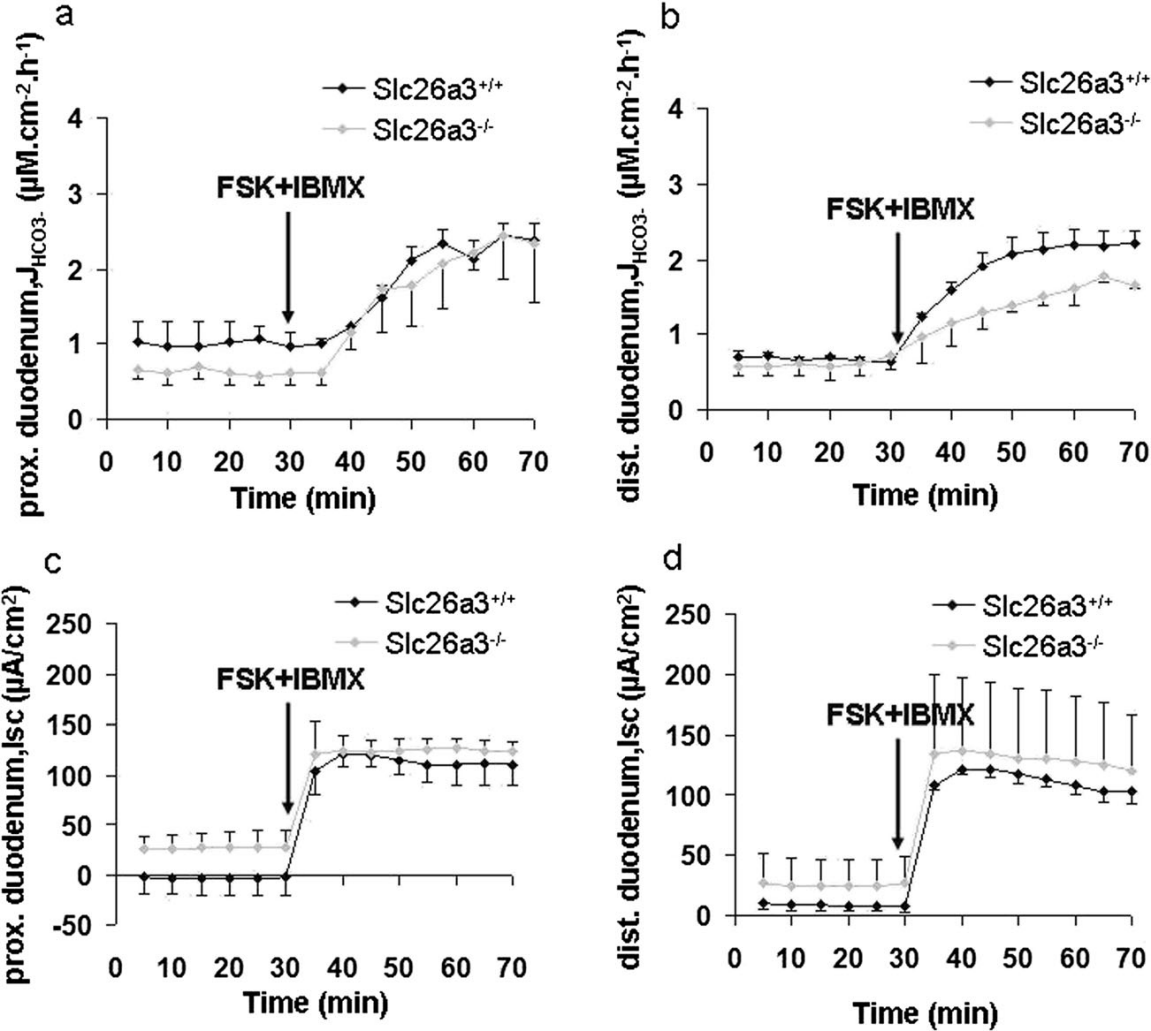
HCO_3_^-^ secretory rate and short-circuit current (I_sc_) of proximal and distal duodenum in Slc26a3^+/+^ and Slc26a3^−/−^ mice. **a**, **c**. The HCO_3_^-^ secretory rate and I_sc_ were identical in the proximal duodenum both at basal state and after FSK + IBMX stimulation between Slc26a3 WT and KO mice. *n* = 5. However, the distal duodenum displayed higher FSK-induced HCO_3_^-^ secretory rate in Slc26a3 WT than KO mice (**b**) but with identical I_sc_ (**d**) *n* = 3–4

The same experiments were performed with isolated proximal and distal duodenal Slc26a3^−/−^ and WT mucosa. As described for in vivo experiments [37], the basal J_HCO3_^-^ but not the FSK-induced ∆J_HCO3_^-^ was reduced in the proximal duodenum (Fig. 9a), whereas we did see a lower ∆J_HCO3_^-^ in distal duodenal mucosa of Slc26a3^−/−^ deficient mice (Fig. 9b). However, no difference was observed in basal I_sc_ and FSK-induced ∆I_sc_ either in proximal or distal duodenum between Slc26a3^+/+^ and Slc26a3^−/−^ mice (Fig. 9c, d).

## Discussion

When first discovered, Slc26a9 was found strongly expressed in the human lung [25]. As detailed in the introduction, we have limited information on the physiological function of Slc26a9 in the airways. Although heterologous Slc26a9 expression influences the transport activity of endogenous CFTR in a variety of airway cell culture systems [6, 26, 29] and a recent publication described the presence of Slc26a9 variants in two patients with bronchiectasis [5], a genome-wide association study revealed an increased incidence of meconium ileus in CF infants with Slc26a9 polymorphisms [41], a susceptibility to CF diabetes [7, 38] but not of severity of CF lung disease [7].

We therefore investigated the distribution of Slc26a9 expression in the human and murine intestinal tract by quantitative real-time PCR and compared it with the expression of the CFTR anion channel in the same segments. In both human and murine intestinal tract, Slc26a9 is expressed very strongly in the stomach and its expression levels decrease abruptly in the duodenum. In the human but not the murine ileum, a specific Slc26a9 amplification product could still be detected, but the expression was extremely low and was absent in the human and murine colon. In contrast, CFTR mRNA expression levels were much lower than those of Slc26a9 in the stomach and much higher in the intestine. The murine liver did not express Slc26a9, while the pancreas displayed weak Slc26a9 expression (Fig. 1).

“Low level” Slc26a9 expression only indicates expression at the whole organ level. In the kidney, for example, neither the original northern analyses nor the PCR amplification gave indication of renal expression of this transporter above background noise [25, 47]. However, the study of Slc26a9 in a renal transport physiology group indicated not only an expression of Scl26a9 in specialized cells of the collecting duct but also a reduction of renal Cl^-^ excretion and high blood pressure in Slc26a9-deficient mice [1]. Thus, even a low level of Slc26a9 expression at the organ level may be functionally highly significant, depending on its cellular location. We therefore performed laser capture microscopy to study the Slc26a9 expression along the duodenal crypt-villus axis. While NHE3, PAT-1, and DRA displayed a villus-predominant expression, CFTR displayed a crypt-predominant expression, as predicted by immunohistochemistry [34]. Slc26a9 was found in the cryptal region. Slc26a9 is thus the first anion transporter besides CFTR with a presumed apical localization and a cryptal expression in the intestine. Since the cryptal region comprises a very minor part of the total cellular mass of the proximal duodenum, a low mRNA expression of a channel protein at the organ level may nevertheless result in significant functional importance of that channel.

Because of the marked gradient of Slc26a9 expression along the duodenal length, we studied fluid transport and HCO_3_^-^ secretion in the murine proximal and distal duodenum separately. Although technically challenging because of the short length, a careful validation of the fluid absorptive measurements indicated that it was possible to detect accurately the microliter changes in fluid content before and after segmental perfusion of the proximal and distal duodenum, even when the perfused intestinal segment was shorter than 1 cm. During isotonic perfusion with a luminal pH of 7.4, the proximal duodenum was in a fluid secretory state, as opposed to the distal duodenum (this paper), as well as the rest of the intestine [10, 36, 46]. In the absence of Slc26a9 expression, we observed a fluid absorptive state in the proximal duodenum in young mice, whereas both Slc26a9 KO and WT mice were in a fluid secretory state in ageing mice. Basal HCO_3_^-^ secretory rate and the HCO_3_^-^ secretory response to FSK were reduced at any age but more so at young age.

Fluid movements are dependent not only on epithelial ion transporters but also on systemic influences such as hydration status, perfusion pressure, and acid–base balance. In addition, the submucosal Brunner’s glands [16] are probably contributing to anion and fluid secretion in the proximal duodenum, based on the expression and dynamic trafficking of ion transporters in these cells [12]. We therefore investigated HCO_3_^-^ secretion and electrophysiological parameters in isolated proximal and distal duodenal mucosa of WT and Slc26a9 KO mice, where the submucosal glands are largely removed in the stripping procedure. For these investigations, the small chamber aperture allows us to study the most proximal part of the duodenum, directly after the pylorus. A significant difference in the basal and FSK-induced HCO_3_^-^ secretory response was again observed between isolated proximal and distal duodenal mucosa. Slc26a9 deletion resulted in a significant decrease in HCO_3_^-^ secretory response in the proximal duodenum; no difference was observed in the distal duodenum.

Explaining the findings on a molecular level based on currently available data may come up with several different models: Firstly, Loriol et al. reported a HCO_3_^-^ conductivity of approximately one eighth of that for Cl^-^ for Slc26a9 expressed in oocytes [26], and Dorwart et al. also reported a low, but discernable, HCO_3_^-^ conductivity of Slc26a9 when expressed in oocytes [14]. We know from isotope flux studies performed on chambered duodenal mucosa from different species in parallel to HCO_3_^-^ titration that the ratio of HCO_3_^-^ to Cl^-^ export into the luminal fluid is between approximately one fifth in the high HCO_3_^-^-secreting rabbit duodenum [40] to approximately one eighth in the rat and mouse ([32] and unpublished observations). Thus, the relatively small increment of HCO_3_^-^ secretory flux could in theory be mediated by Slc26a9 itself. If that were the case, we might expect to see an eightfold higher effect of Slc26a9 deletion on the basal I_sc_, which was not observed. Therefore, we consider it unlikely that the higher HCO_3_^−^ secretory rate in Slc26a9 WT duodenum is due to the (presumably very low) HCO_3_^−^ permeability of Slc26a9 itself.

**Fig. 10:**
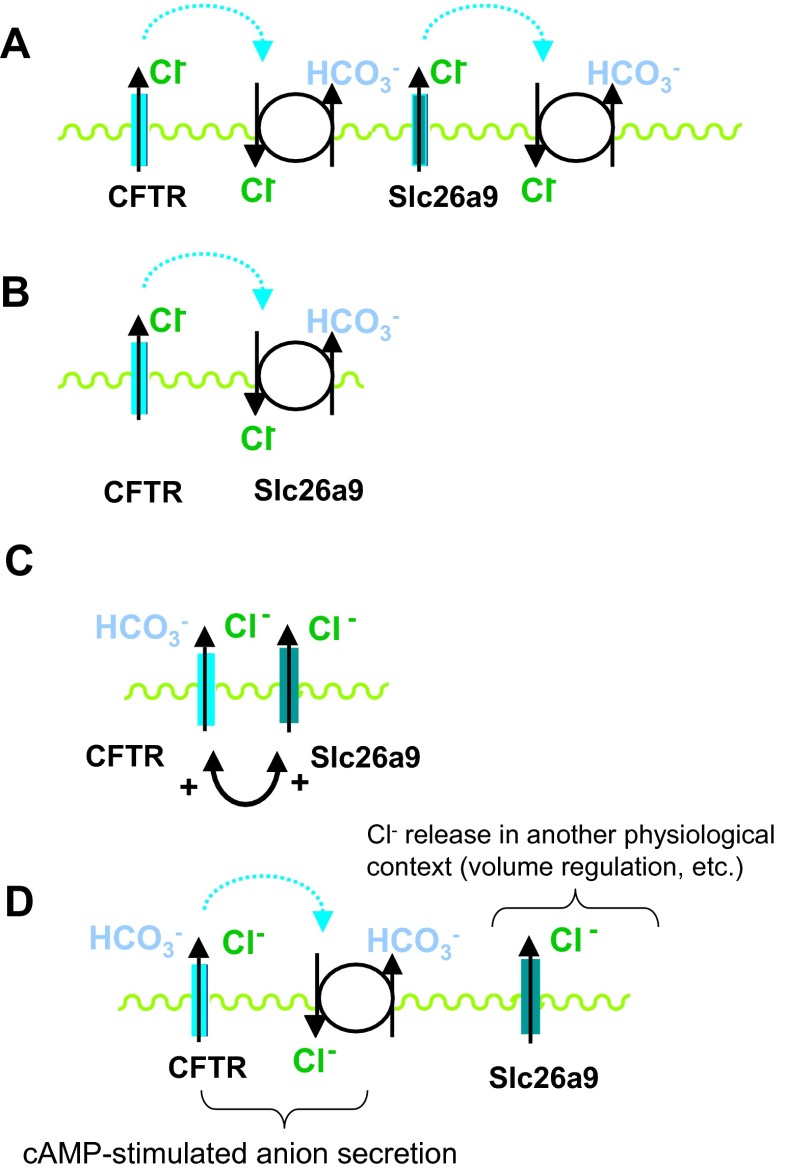
Schematic diagrams depicting four potential modes of Slc26a9 function enhancing proximal duodenal HCO_3_^−^ secretion. **a** Slc26a9 is a chloride channel and facilitates the Cl^−^ recycling via another Cl^−^/HCO_3_^−^ exchanger. This function would be similar to that proposed for the CFTR channel. While no cryptal expression for Slc26a6 and Slc26a3 has been described, an overlap of expression of Slc26a9 and the other apical Slc26a members with an unequivocal Cl^−^/HCO_3_^−^ exchanger function at the base of the villi is feasible. **b** Slc26a9 itself functions as the anion exchanger that is functionally coupled to CFTR. This model would explain the HCO_3_^−^ secretion data but not the fluid absorption data. In addition, the majority of heterologous expression studies define Slc26a9 as a Cl^−^ channel. **c** CFTR and Slc26a9 interact structurally and/or functionally in a positive fashion. Several different mechanisms can be envisioned how this could come about (see “Discussion”). **d** Slc26a9 is a crypt enterocyte anion channel that has functions not directly related to transepithelial anion transport but possibly involved in volume control/apoptosis/migration/differentiation. In this case, the altered ion transport would be a secondary phenomenon based on changes in cellular growth and differentiation patterns

Secondly, Slc26a9 could, in theory, be an anion exchanger working in parallel with CFTR, in a classical “Cl^−^ recycling” fashion. This would best explain our observations, but the majority of heterologous expression data as well as our study in the lung do not support this conclusion [14, 26–28].

Another frequently discussed molecular model of Slc26-CFTR interactions envisions an interaction of the STAS domain of the Slc26 members with the regulatory domain of CFTR. Bertrand et al. have described a reciprocal stimulation of both Slc26a9 as well as CFTR current by STAS-R domain interaction in a protein kinase A (PKA)-dependent fashion [6], similar to what has been described for the interaction between the anion exchangers Slc26a3 and Slc26a6. Avella has observed an enhanced CFTR processing and membrane insertion in heterologous expression systems [4]. Ousingsawat et al. found that an inhibition of endogenous Slc26a9 expression in a heterologously CFTR-expressing bronchial cell line decreased the UTP- as well as the cAMP-induced anion current but only in a polarized cell line, whereas Slc26a9 coexpression inhibited WT CFTR currents expressed in HEK cells [29]. An inhibition of the Slc26a9 STAS domain was also observed by Chang et al. in Xenopus oocytes, whereas a chimera with the Slc26a6 STAS domain stimulated the currents [9]. Our findings in the proximal duodenum are consistent with previous reports on a positive interaction between CFTR and Slc26a9, at least regarding the higher FSK-induced HCO_3_^-^ secretory response in Slc26a9 WT duodenum [27]. However, no significant difference was observed in the I_sc_ response to FSK between WT and Slc26a9 KO mucosa, and the fluid secretory response of the duodenum of Slc26 KO appeared normal. Neither parameter accurately reflects CFTR activity in the native intestine, where the rate-limiting steps for anion secretion may be the uptake/generation of the anions [11, 15, 19, 42].

The results resemble those described by Walker et al. in the DRA-deficient duodenum, who found a reduction in basal and FSK-induced HCO_3_^-^ secretion but not in I_sc_ response [43]. In our studies, the DRA-deficient-isolated proximal duodenal mucosa did not display a reduction in basal J_HCO3_^-^ and FSK-induced ∆J_HCO3_^-^ compared to WT littermates, whereas the distal duodenal mucosa had lower HCO_3_^-^ secretory rates in DRA-deficient mucosa (Fig. 9a–b). Given the fact that DRA is also expressed in the proximal duodenum but in a villus-predominant expression (Fig. 2b), the data are not easy to explain. It is possible, but has not been investigated, that the interaction with Slc26a9 alters the HCO_3_^-^ permeability of CFTR, or vice versa. It is thus feasible that in the proximal duodenum, Slc26a9 and CFTR interact in a positive fashion, whereas in the distal duodenum, the anion exchanger(s) Slc26a3 (and possibly Slc26a6) perform this task. It is of note that while Slc26a3 and Slc26a6, as well as CFTR, are expressed at much higher levels than Slc26a9 in the duodenum, their cellular location is not identical for the majority of Slc26a3 and Slc26a6 with that of CFTR and Slc26a9. Since for the STAS-R domain interaction, a 1:1 molar ratio of the transporters is envisioned, it is clear that duodenal CFTR in the crypts does not find abundant Slc26a3 and/or Slc26a6 for interaction. Thus, the Slc26a9-CFTR interaction may be of particular physiological significance in this location. If CFTR preferentially interacts with an anion conductance in the proximal duodenum but with an anion exchanger in the distal duodenum, this may explain why the WT proximal duodenum is in a fluid secretory state and the distal duodenum in a fluid absorptive state. This concept is corroborated by the fact that Slc26a9 deletion results in a fluid absorptive proximal duodenum in young mice.

Another possibility to explain the higher basal HCO_3_^−^ output in the proximal duodenum could be a functional interaction of Slc26a9-mediated Cl^−^ efflux and Slc26a3-mediated Cl^−^ uptake in exchange for HCO_3_^−^. However, there is little evidence for coexpression of the two transporters in the same cells in the proximal duodenum. But still, an overlap zone may well exist. The different potential modes of Slc26a9-mediated enhanced anion transport rates in duodenal crypt cells are depicted in the cartoons displayed in Fig. 10a–d; however, the speculative nature of these cartoons has to be kept in mind.

A surprising finding of this study was the enhanced proliferative status of the proximal duodenum in the young Slc26a9 KO mice, with lengthening of both the crypts and the villi. A similar observation has been previously made in CFTR-deficient duodenum [17]. The establishment of an enteroid culture system ex vivo from the small intestine of CFTR WT and KO mice enabled the group of Lane Clarke to study crypt cell proliferation in isolated CFTR-deficient epithelium in the absence of systemic factors from the host [23]. They found that CFTR-deficient crypt cells had a more alkaline pH_i_, which caused alterations in the WNT/β-catenin signaling [22]. We currently do not know whether Slc26a9-deficient crypt cells also have more alkaline pH than WT crypt cells. Another factor that may influence proliferation is a potential effect of Slc26a9-mediated channel activity on cell volume. A constitutively active anion channel is supposed to decrease both pH_i_ and cell volume, thus having an inhibitory effect on proliferation [21].

The Slc26a9 KO mice develop hypergastrinemia with ageing. We did find not find evidence in the literature that hypergastrinemia cause duodenal crypt and villus elongation, or that duodenal crypt cells possess the CCK-B/gastrin receptor, or that they are more sensitive to gastrin at young age of the mice, or that the effect of gastrin in the duodenum is restricted to the proximal duodenum. In addition, KCNQ1 KO mice also had massive hypergastrinemia and a loss of acid secretory capacity from birth [39] but no duodenal hyperproliferation (supplementary Fig. 3a, b). Nevertheless, indirect sequelae of the altered function of the gastric epithelium on the proximal duodenum of young mice cannot be ruled out.

How do we reconcile our findings of altered ionic transport in the proximal duodenum of Slc26a9 KO mice with an increased incidence of meconium ileus in CF infants having polymorphisms in the *Slc26a9* gene? Our data clearly show that Slc26a9 expression is negligible in the part of the murine and human intestine that is obstructed in the murine CFTR-deficiency-related intestinal obstruction (mid jejunum to ileum) and human CF meconium ileus (ileum and proximal colon); and Slc26a9 expression is not increased in CFTR deficient compared to WT distal small intestine and colon (data not shown). Nevertheless, the additional deletion of Slc26a9 in CFTR KO mice caused a massive decrease in survival during and after the weaning phase, whereas Slc26a9 KO mice had a high perinatal death rate, most likely due to pulmonary complications [24], but had a survival rate from the time of weaning that was not different from that of WT littermates. Although we could not ascertain the cause of death in most of the Slc26a9/CFTR double knockouts, because they have not been found early enough before being eaten by the mothers, we know from previous observations in our CFTR-deficient mouse colony, as well as of the present CFTR-deficient colony, that the postweaning death is virtually always due to intestinal obstruction (unless caused by damage from parents/siblings or technical accidents with the mouse breeding). Thus, we are confident that the strongly increased incidence of death during the weaning phase in the Slc26a9/CFTR double KO mice, despite intense nutritional treatment and PEG-containing drinking fluid, is also related to intestinal complications. Therefore, Slc26a9 deficiency is a prominent risk factor for early death in CFTR-deficient mice, probably due to intestinal obstruction. This finding also strengthens (but does not prove) the assumption of a positive functional Slc26a9-CFTR interaction.

We therefore believe that the reason for increased intestinal obstructions in CF mice or humans with the absence or malfunction of Slc26a9 is the upper GI tract dysfunction resulting in maldigestion and impaired downstream signaling. The severe gastric phenotype has been previously described [48]. Another previous study demonstrated that *Slc26a9*-deficient mice had a virtually absent HCO_3_^-^ secretory response to luminal acid, while that to FSK was intact [37]. That study demonstrated that the downstream signaling pathways between acid-induced and FSK-induced duodenal HCO_3_^-^ secretion only partially overlap at the level of the epithelium [37], and another previous study shows that approximately 70 % of the acid-induced HCO_3_^-^ secretory response is mediated by a vagal reflex whereas the FSK-induced HCO_3_^-^ secretory response is not affected by vagotomy [35]. Firstly, this suggests that Slc26a9 may be regulated by as yet unknown signaling pathways. Secondly, it suggests that the duodenal-pancreatic axis (the sensing of the intraluminal environment, i.e., pH causing the output of hormones and neurotransmitters that affect pancreatic secretions) may also be affected by Slc26a9 deletion.

In summary, our study shows that even a low Slc26a9 expression level can cause marked decrease in epithelial HCO_3_^-^ secretion and fluid transport, particularly at young age. Slc26a9 deletion strongly reduces the survival of CFTR KO mice, particularly in the weaning phase, where the mice die of intestinal obstruction. Slc26a9 is not only expressed in the stomach and proximal duodenum in mouse and humans, but also to some extent in the pancreas, at similar expression levels to those in the distal duodenum. It is not expressed at significant levels in those intestinal segments that actually experience the obstructions (distal small intestine in mice, distal small intestine and proximal colon in humans). Our data suggest that the higher risk of meconium ileus in CF infants with Slc26a9 polymorphisms is due to upper intestinal dysfunction including the stomach, the duodenum, and possibly the pancreas. We do not yet know the cellular location of pancreatic and biliary tract Slc26a9, but if it is in the ductal epithelia, the effect on biliary and pancreatic HCO_3_^-^ secretion may be similar to that in the duodenum. Future studies in biliary and pancreatic secretion of *Slc26a9*-deficient mice may add to our understanding why polymorphisms in this gene are risk factors not only for meconium ileus in CF infants, but also for CF diabetes.

## Electronic supplementary material

Below is the link to the electronic supplementary material.

ESM 1 (DOC 261 kb)

## Abbreviations

CFTR: Cystic fibrosis transmembrane conductance regulator
CF: Cystic fibrosis
cAMP: Cyclic adenosine monphosphate
DRA (Slc26a3): Downregulated in adenoma
FSK: Forskolin
GI: Gastrointestine
I_sc_: Short-circuit current
IBMX: 3-Isobuty1-1-methlxanthine
J_HCO3_^−^: Bicarbonate secretory rate
Slc26a9: Solute carrier family 26 member 9
