# Supplementary material for: Loss of Slc26a9 anion transporter alters intestinal electrolyte and HCO3- transport and reduces survival in CFTR-deficient mice
Source: Pflugers Arch. 2014 Jun 27;467(6):1261–75. doi: 10.1007/s00424-014-1543-x (PMC4434866; doi:10.1007/s00424-014-1543-x)
Supplement: Supplementary file 1 — (DOC 261 kb) [file 424_2014_1543_MOESM1_ESM.doc]

**Loss of Slc26a9 anion transporter alters intestinal electrolyte and HCO_3_^‑^ transport and reduces survival in CFTR‑deficient mice**

**Pflügers Archiv**

Xuemei Liu^1*^, Taolang Li^1,2*^, Brigitte Riederer^1^, Henrike Lenzen^1^, Lisa Ludolph^1^, Sunil Yeruva^1^, Biguang Tuo^3^, Manoocher Soleimani^4^, Ursula Seidler^1^

^1^Dept. of Gastroenterology, Hannover Medical School, Hannover, Germany, ^2^Department of Gastrointestinal Surgery and ^3^Gastroenterology, Affiliated Hospital of Zunyi Medical College, Zunyi, P.R.China, ^4^Center on Genetics of Transport and Epithelial Biology, University of Cincinnati, Cincinnati, Ohio, United States of America

*these authors contributed equally and share first authorship

Author for correspondence: Prof. Dr. U. Seidler, Department of Gastroenterology, Hepatology and Endocrinology, Hannover Medical School, Carl‑Neuberg‑Straße 1, D‑30625 Germany, Tel.: +49‑511‑532‑9427; Fax No. +49‑511‑532‑8428, email: Seidler.Ursula@mh‑hannover.de

**Supplementary Table 1: List of Primer Sequences**

| **Genotyping** | **Sequence** | **Product length, Accession No.** |
| --- | --- | --- |
| Slc26a9.for | 5´-TCG TTC TTC CTG AGC CTG CC-3´ | 100bp, NM_177243.3 |
| Slc26a9.rev | 5´-CCA GTG TGG AGC CAT TTC GA-3´ |  |
| CFTR.for | 5´- TTC TTC ACG CCC CTA TGT CGA-3´ | 145bp, NM_021050 |
| CFTR.rev | 5´- GCT CCA ATC ACA ATG AAC ACC A-3´ |  |
| DRA.for | 5´- TTC CCC TCA ACA TCA CCA TCC-3´ | 110bp, NM_021353 |
| DRA.rev | 5´- GTA AAA TCG TTC TGA GGC CCC-3´ |  |
| NHE3.for | 5´- AGG CCA CCA ACT ATG AAG AG-3´ | 110bp, NM_00108160 |
| NHE3.rev | 5´- AGG GGA GAA CAC GGG ATT ATC-3´ |  |
| PAT-1.for | 5´- GGC TCC TGG GTG ATC TGT TA-3´ | 100bp, NM_134420 |
| PAT-1.rev | 5´- CCA AAC ATA GGA GGC AAT CC-3´ |  |
| Actin. for | 5´-AGA GGG AAA TCG TGC GTG AC-3´ | 138bp,NM_007393.2 |
| Actin.rev | 5´-CAA TAG TGA TGA CCT GGC CGT-3´ |  |

The details of primer sequence, product length and the accession number of different genes that we used.


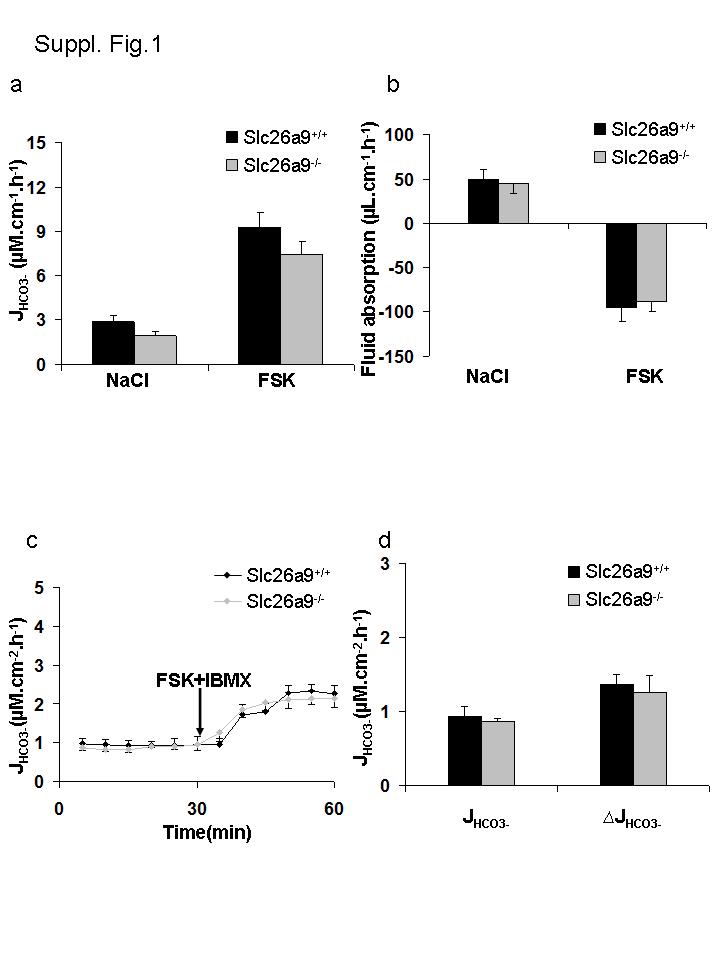


**Supplementary Fig 1: No difference in HCO_3_^‑^ secretion as well as fluid absorption in the distal duodenum between Slc26a9 WT and KO mice**

(a) and (c) shows the identical HCO_3_^‑^ secretion of distal duodenum between Slc26a9 WT and KO mice both *in vivo* and *in vitro* as well as similar fluid absorption (b). (d) shows the J_HCO3-_ and FSK-induced ∆J_HCO3-_ calculated form each experiment individually. n=5‑6.


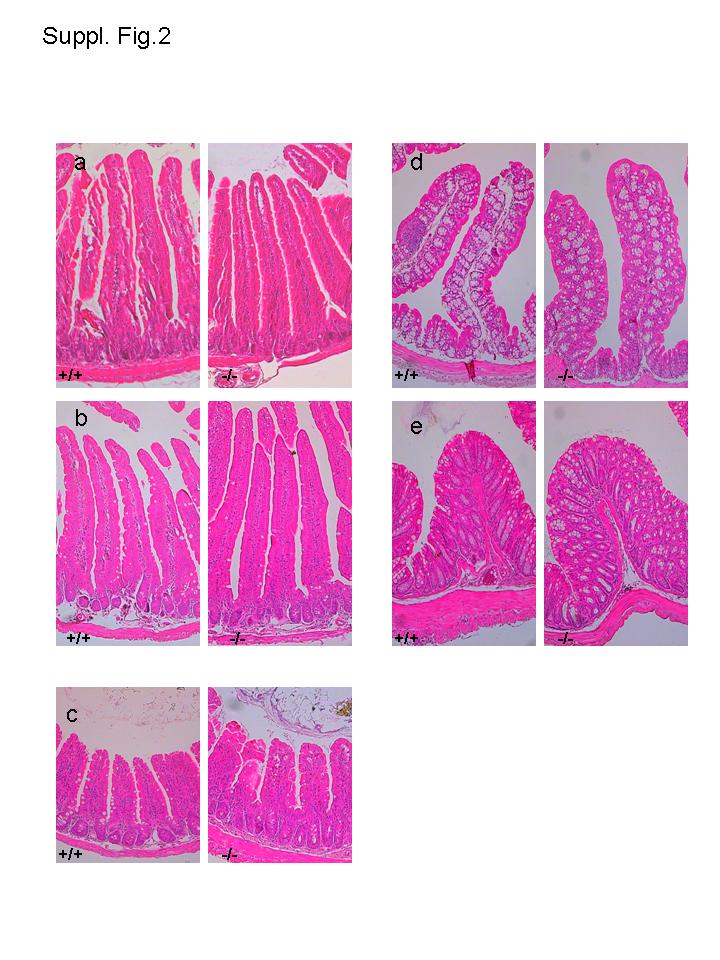


**Supplementary Fig 2: Morphology of different segments of intestine in *slc26a9* null mice**

No obvious differences were observed in other segments of intestine in Slc26a9^+/+^ and ^‑/‑^ mice. (a) distal duodenum, (b) jeunum, (c) ileum, (d) proximal colon, (e) distal colon. Scale bar: 200µm, n=4‑5.


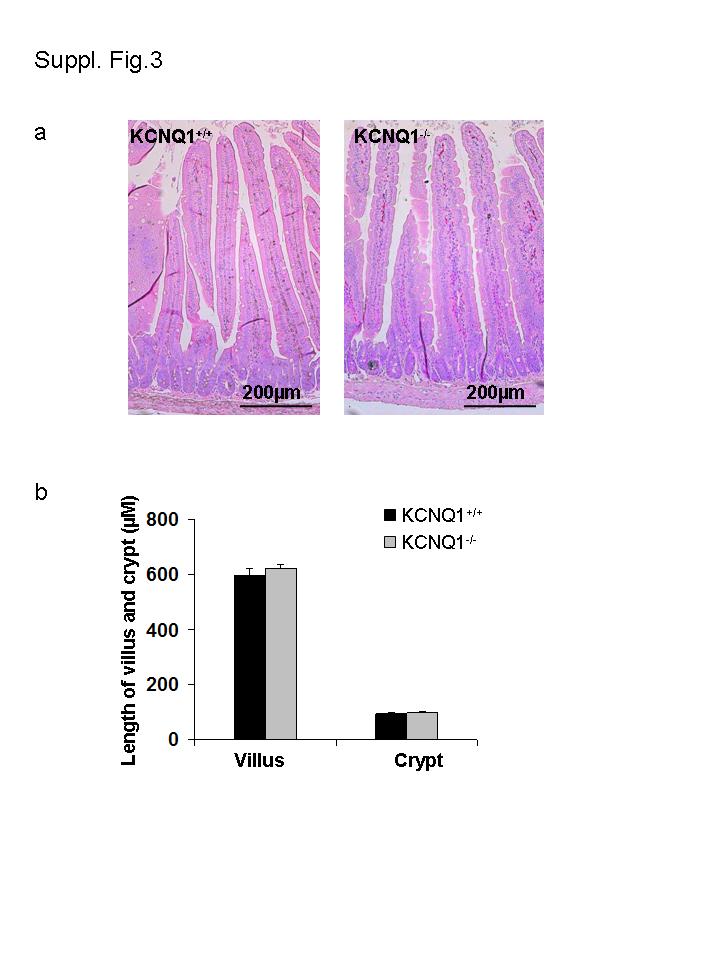


**Supplementary Fig 3: Histological analysis in the proximal duodenum of *kcnq1* deficient mice**

(a-b) The length of the villi and crypts were similar in proximal duodenum of KCNQ1 WT and KO mice, although these mice also display no spontaneous or stimulated acid secretion and marked hypergastrinemia [39]. n=3‑4. Scale bar: 200µm
